# Clinical and Therapeutic Insights into Sepsis: A Retrospective Observational Study of Inflammatory Markers, and Outcomes

**DOI:** 10.3390/biomedicines13102566

**Published:** 2025-10-21

**Authors:** Dragoș Ștefan Lazăr, Adina-Alexandra Nanu, Ilie-Andrei Condurache, Casandra Bulescu, Catrinel Tudosie, Alexandra Ioana Grigore, Corneliu Petru Popescu, Simin Aysel Florescu

**Affiliations:** 1 Department of Infectious Diseases, “Carol Davila” University of Medicine and Pharmacy, 020021 Bucharest, Romania; dragos.lazar@umfcd.ro (D.Ș.L.); casandra.sapoiu@rez.umfcd.ro (C.B.); catrinel.gurgui@rez.umfcd.ro (C.T.); corneliu.popescu@umfcd.ro (C.P.P.); simin.florescu@umfcd.ro (S.A.F.); 2 “Dr Victor Babes” Clinical Hospital of Infectious and Tropical Diseases, 030303 Bucharest, Romania; ilie.condurache@spitalulbabes.ro (I.-A.C.); ioana.grigore@spitalulbabes.ro (A.I.G.)

**Keywords:** sepsis, neutrophil-to-lymphocyte ratio, inflammatory biomarkers

## Abstract

**Introduction:** Sepsis is a life-threatening condition caused by dysregulated host responses to infection, leading to organ failure and high mortality. Early recognition, especially in vulnerable populations, remains challenging due to variable presentations. Key biomarkers like CRP, procalcitonin, fibrinogen, and neutrophil-to-lymphocyte ratio (NLR) aid in diagnosis, monitoring, and prognosis. Rapid identification and targeted therapy are critical, particularly amid rising antimicrobial resistance. This study aims to analyze the relationship between early biomarker levels and patient outcomes, focusing on mortality risk prediction within the first week of hospitalization. **Methods:** A retrospective study of 198 sepsis patients hospitalized in Bucharest, Romania, between January and December 2024, analyzing inflammatory biomarkers at admission—T0, 48–72 h—T1, and one week—T2, to identify predictors of clinical outcomes. **Results:** In patients under 65 years old, fibrinogen, CRP, and NLR significantly decreased from T0 to T2, especially in survivors. In contrast, patients over 65 years old showed less consistent biomarker changes, with higher mortality associated, with comorbidities such as heart failure and cancer. Overall, early reductions in inflammatory markers correlated with better outcomes, highlighting their prognostic value in sepsis management. **Conclusions:** In sepsis patients over 65 years old, a stable or rising neutrophil-to-lymphocyte ratio (NLR) and fibrinogen levels after the first week of hospitalization may indicate a poor prognosis, whereas decreasing levels suggest a better chance of survival.

## 1. Introduction

Sepsis is a life-threatening condition characterized by a dysregulated host response to an infectious trigger, leading to multiple organ dysfunction and high mortality rates. It is estimated to account for approximately 11 million deaths annually worldwide [1,2].

The clinical definition of sepsis has undergone significant evolution over the past three decades, reflecting advancements in our understanding of its pathophysiology. In 1991, the American College of Chest Physicians and the Society of Critical Care Medicine introduced the first consensus definition, characterizing sepsis as a systemic inflammatory response syndrome (SIRS) due to infection, with criteria including abnormal temperature, heart rate, respiratory rate, and white blood cell count. This was refined in 2001 (Sepsis-2), which maintained the SIRS criteria but expanded the list of clinical signs and symptoms to reflect bedside observations better. However, limitations in sensitivity and specificity led to the development of the Sepsis-3 definition in 2016, which defines sepsis as life-threatening organ dysfunction caused by a dysregulated host response to infection. Organ dysfunction is identified by an increase in the Sequential Organ Failure Assessment (SOFA) score of 2 points or more. Despite its improved prognostic value, Sepsis-3 still presents challenges in early recognition, particularly in elderly or immunocompromised patients [1,3,4,5].

Looking ahead, future definitions may incorporate dynamic biomarkers, molecular signatures, and AI-driven diagnostic models to better reflect the heterogeneity of sepsis. Initiatives such as SPARK (sustained by Stanford University) and SENECA (Sepsis ENdotyping in Emergency Care) aim to redefine sepsis as a spectrum of biologically distinct subtypes, which could enable more targeted and timely interventions [6].

While sepsis can affect anyone, certain populations are at increased risk, including the elderly, infants, pregnant women, and immunocompromised patients of any age [1]. The clinical presentation of sepsis is highly variable and can often be misleading in its early stages. This poses significant challenges for differential diagnosis, particularly in patients with pre-existing comorbidities [7]. Common signs of sepsis include fever, tachycardia, hypotension, rapid respiratory rate, and altered mental status. Without prompt recognition and intervention, these can progress to septic shock, multi-organ failure, and ultimately death [1].

Sepsis commonly arises from respiratory tract infections (especially pneumonia), followed by urinary, abdominal, hepatobiliary, and soft tissue infections [8]. The primary infection source often correlates with severity; for example, pneumonia-associated sepsis is linked to higher ICU admission rates and worse outcomes [8]. In most cases, sepsis is of bacterial origin, making the rapid initiation of antibiotic therapy critical to improving patient outcomes and survival [9].

Depending on the source, sepsis may result from pathogens acquired in the community or within healthcare settings, with the latter representing a significant challenge for modern medicine in the 21st century [10].

Sepsis not only poses a clinical challenge due to its severity and complexity but also because of the growing burden of antimicrobial resistance [11]. Treating sepsis caused by multidrug-resistant organisms remains one of the most pressing issues in contemporary medicine. It carries substantial implications for patient outcomes, hospital microbiota, healthcare costs, and mortality rates [11].

Therefore, rapid identification, appropriate antibiotic therapy, and prognostic biomarker monitoring are key to improving outcomes. Over time, several potential biomarkers have been researched to characterize this complex syndrome. Although none of them can perform perfectly in all encountered situations, a few have become established in current practice. Inflammatory markers such as C-reactive protein (CRP), procalcitonin (PCT), and fibrinogen are routinely used to evaluate infection severity and therapeutic response [12,13]. Inflammatory biomarkers such as C-reactive protein (CRP), procalcitonin (PCT), and fibrinogen play a pivotal role in the early diagnosis, monitoring, and prognostic assessment of sepsis. CRP is an acute-phase protein synthesized in the liver in response to interleukin-6 (IL-6) and tumor necrosis factor-alpha (TNF-α) during systemic inflammation [14]. PCT, a precursor of the hormone calcitonin, is produced by extrathyroidal tissues—especially hepatocytes and monocytes—during bacterial infections, under the stimulation of IL-1β, IL-6, and bacterial endotoxins [15]. Fibrinogen, also synthesized in the liver, is not only a marker of inflammation but also contributes to coagulopathy often seen in septic patients [16]. In recent years, meta-analyses have confirmed that the Neutrophil-to-Lymphocyte Ratio (NLR) is a valuable prognostic marker in sepsis, offering quick, cost-effective insights into disease severity and supporting clinical decision-making [17,18].

The dynamic changes in these markers provide crucial information regarding the host response and treatment effectiveness. A declining trend in CRP and PCT levels within the first 48–72 h after initiating therapy is generally associated with better outcomes, while persistently elevated or rising levels suggest ongoing infection, treatment failure, or a complicated course [13,19]. CRP typically rises within 6–8 h of the inflammatory stimulus and peaks at 48 h, while PCT rises within 2–4 h and peaks earlier, often within 24 h [15]. Fibrinogen increases more gradually and may remain elevated longer, reflecting both inflammation and coagulation activation [18]. Monitoring the trajectory of these biomarkers allows clinicians to assess disease progression, guide de-escalation of antibiotic therapy, and predict complications such as septic shock or multi-organ dysfunction. Their integration into clinical decision-making enhances risk stratification and individualized patient management [12].

The main objective of this study is to analyze the associations between inflammatory biomarker levels, assessed within the first seven days of hospitalization, and patient outcomes, specifically comparing mortality rates versus survival, in order to identify potential prognostic indicators.

## 2. Materials and Methods

A retrospective analysis was conducted on 198 patients diagnosed with sepsis and admitted to the “Dr. Victor Babeș” Clinical Hospital of Infectious and Tropical Diseases (VHB) in Bucharest, Romania, a tertiary care institution, in the period from 1 January to 31 December 2024. Patients were identified based on diagnostic codes recorded at discharge, regardless of outcome, using the ICD-10-CM classification A41. Diagnoses were established by the attending physicians in line with hospital protocol, based on clinical findings and ancillary data (laboratory and, where available, imaging), and in accordance with qSOFA/SOFA criteria. Individuals with hospital stays of fewer than seven days were excluded following preliminary data screening to ensure consistency in evaluating the evolution of inflammatory biomarkers and treatment outcomes.

### 2.1. Hospital Protocol Summary for Sepsis Management

At our institution, the evaluation of patients upon admission follows a standardized protocol that includes a comprehensive panel of laboratory tests. For the purposes of this study, the key parameters analyzed were: white blood cell count (WBC), C-reactive protein (CRP) with normal values below 0.5 mg/dL (immunonephelometry, Cardio Phase^®^ hsCRP, Siemens, Munich, Germany, Catalog no.: OQIY21), fibrinogen, with normal values less than 400 mg/dL (Clauss method, Coagulometer STA Compact Max3, Stago, Reagent: STA LIQUID FIB, manufacturer Stago, Asnières-sur-Seine, France, reference number Ref. 00673), and, when available, procalcitonin (PCT), with normal values less than 0.05 ng/mL (chemiluminescent microparticle immunoassay, ALINITY/Abbott, PCT Reagent Kit, Chicago, IL, USA, Catalog no.: 01R1822). These tests are routinely used to assess the inflammatory response and aid in the early identification of sepsis.

In all suspected cases of infection, blood cultures are systematically obtained prior to the administration of the first dose of antibiotics. Indications for blood culture collection include fever ≥ 38 °C, hypothermia ≤ 35.5 °C, shivering, or even the absence of fever (afebrile presentation). Ideally, two sets of blood cultures are collected, each consisting of two aerobic and two anaerobic bottles. However, in cases where full sets are not feasible due to the limited availability of blood culture bottles, a single set is collected.

Empirical antibiotic therapy is initiated immediately after culture collection, based on suspected infection site and local resistance patterns, and adjusted as needed once microbiological data becomes available. Common empirical regimens include carbapenems, glycopeptides, and beta-lactam/beta-lactamase inhibitor combinations, with de-escalation performed upon clinical or microbiological improvement. Supportive care measures like fluid resuscitation, vasopressors, and oxygen are applied as needed.

This protocol ensures uniformity in diagnostic and therapeutic approaches, enabling reliable data collection for evaluating inflammatory markers, treatment response, and patient outcomes.

### 2.2. Bacterial Identification and Antibiotic Susceptibility

Bacterial species identification was conducted using automated systems based on chemical principles, specifically, the exoenzymatic properties of bacteria (VITEK 2C), as well as physical principles through mass spectrometry techniques, namely MALDI-TOF (Matrix-Assisted Laser Desorption/Ionization—Time of Flight Mass Spectrometry) (BRUKER). Antibiotic susceptibility was assessed using disk diffusion, MIC determination with VITEK 2C, E-test, and broth microdilution (EUCAST standard). The detection of carbapenemase producers was performed with rapid immunochromatographic tests, confirmed by molecular methods like RT-PCR and ESBL production was identified using the double-disk synergy test. All testing and interpretation adhered to EUCAST guidelines.

### 2.3. Data Collection and Statistical Analysis Protocol

Data were collected on demographics, comorbidities, infection sources, microbiological findings, inflammatory biomarkers at three time points—T0 (admission), T1 (48–72 h), and T2 (days 7–8), as well as treatment regimens, and clinical outcomes. We selected these time points (T1 and T2) because we believe that evaluation at 48–72 h reflects the effect of initial (empiric) treatment, while assessment after one week also incorporates the results of targeted therapy based on bacterial identification and antibiogram analysis.

Statistical analysis was conducted using IBM SPSS Statistics version 25. Qualitative variables were analyzed using Fisher’s Exact Test or Pearson’s Chi-Square Test. For quantitative variables, normality was assessed via the Shapiro–Wilk test. Depending on distribution, comparisons were made using the Friedman test (with post hoc Dunn–Bonferroni tests) or Repeated-Measures One-Way ANOVA (with Greenhouse–Geisser correction when needed according to the Mauchly test for Sphericity, along with post hoc Bonferroni tests) for related samples, and the Mann–Whitney U Test or Student’s *t*-Test for independent samples. A significance level of α = 0.05 was used throughout.

### 2.4. Ethical Considerations

In accordance with Romanian regulations, all patients admitted to the hospital were given the opportunity to provide or decline consent for participation in research activities, as part of the administrative paperwork completed at admission. For this study, only patients who explicitly consented to the use of their anonymized data for research purposes were included. All data were handled in compliance with institutional ethical standards and national data protection laws. The study was approved by the local ethics committee of the “Dr. Victor Babeș” Clinical Hospital No. 10071/05.06.2025.

## 3. Results

The 198 patients hospitalized in 2024 with a diagnosis of sepsis had the following characteristics, which are presented in Table 1.

The characteristics of the patient cohort are summarized as follows: the majority were female (50.8%) with a mean age of 65.05 ± 17.23 years (most of the patients having over 64 years—62.3%), predominantly residing in urban areas (81.9%). A minority (6.6%) were institutionalized, while over half (51.5%) had multiple hospitalizations. Common comorbidities included diabetes mellitus (28.1%), chronic heart failure (41.9%), and neurological conditions (22.6%).

The primary sources of infection were respiratory (30.1%), urinary (21%), and gastrointestinal (19.9%). A percentage of 11.8% had mixed origins. The predominant infecting agents were Gram-negative bacilli (46.5%). Although all pathogen types were identified using standardized laboratory methods, for simplicity, the bacteria in Table 1 were grouped solely by Gram stain affinity, and the identified fungi were not presented separately. Immunosuppression was identified in 28.6%, and antibiotic resistance was present in 28.7%, mainly due to carbapenemases in Gram-negative bacilli.

At admission (T0), most patients received carbapenems, with 105 cases (52.8%), followed by glycopeptides in 83 cases (41.7%) and cephalosporins in 63 cases (31.7%). The average duration of treatment at this stage was approximately 8.72 days, with a median of 7 days. Antifungal therapy was administered to 20.2% of patients, and corticosteroids were used in 21.2% of cases, with an average duration of about 9 days.

Eighteen percent of the patients had received antibiotic treatment before hospitalization. The mean length of hospitalization was 14.7 ± 10.39 days. ICU admission occurred in 13.5%, and the mortality rate at discharge was 18.6%.

Laboratory parameters at T0 showed elevated levels of inflammatory markers: mean leukocytes were 13,659 ± 11,108/μL (median 11,900), neutrophils 11,288 ± 9012/μL (median 9500), and lymphocytes 1548 ± 5545/μL (median 800). Fibrinogen averaged 622 ± 226 mg/dL, CRP 16.55 ± 10.97 mg/L, and procalcitonin 9.36 ± 16.3 ng/mL, with median values indicating significant variability.

We compared the leukocyte, neutrophils, and lymphocyte counts (WBCs and subsets), and the neutrophil-to-lymphocyte ratio (NLR) from patients’ complete blood counts at the T0, T1, and T2 time points evaluated in our study (Table 2 and Table 3).

The results from Table 2 show significant changes for all investigated parameters. Post hoc tests show a significant decrease in leukocytes/neutrophils count/NLR from T0 to T1 (*p* < 0.001/*p* < 0.001/*p* < 0.001), T0 to T2 (*p* < 0.001/*p* < 0.001/*p* < 0.001) with no significant changes from T1 to T2 in leukocytes and neutrophils (*p* = 1.000/*p* = 0.292) and a significant decrease in NLR from T1 to T2 (*p* < 0.001) while a statistically significant increase in lymphocyte count was observed from T0 to T1 (*p* = 0.003), T0 to T2 (*p* < 0.001), and T1 to T2 (*p* < 0.001).

**Figure 1:**
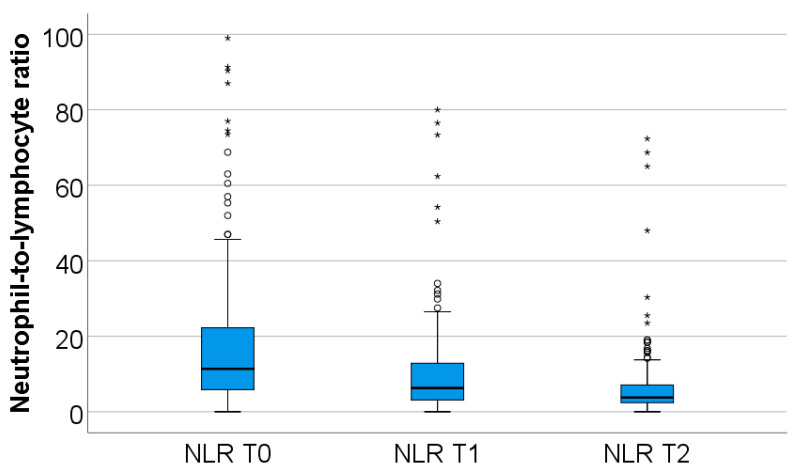
Dynamics of NLR T0–T2. NLR = neutrophil-to-lymphocyte ratio. T0, T1, and T2 = Time 0, 1, and 2. (*) = outliers values.

The dynamics of neutrophil-to-lymphocyte ratio values from T0 to T2, observed in the study group, are shown in Figure 1.

In the study group, neutrophil values decline during the first week of hospitalization (from T0 to T2), while lymphocyte values increase over the same period. Both changes were highly statistically significant (*p* < 0.001).

**Figure 2:**
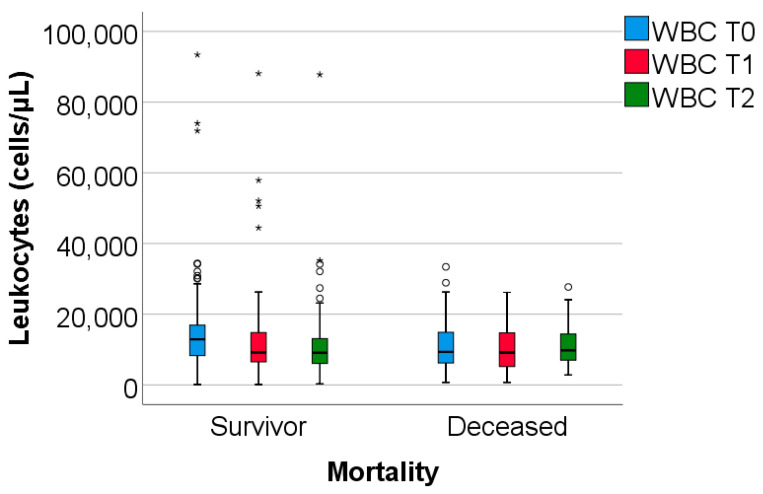
The dynamics of WBC were observed at T0, T1, and T2. WBC = white blood cells. T0, T1, and T2 = Time 0, 1, and 2. (*) = outliers values.

**Figure 3:**
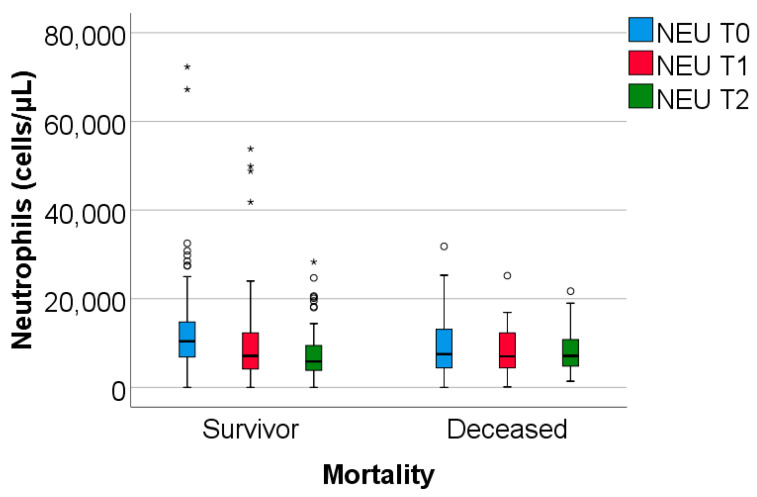
The dynamics of neutrophils were observed at T0, T1, and T2. NEU = Neutrophils. T0, T1, T2 = Time 0, 1, and 2. (*) = outliers values.

**Figure 4:**
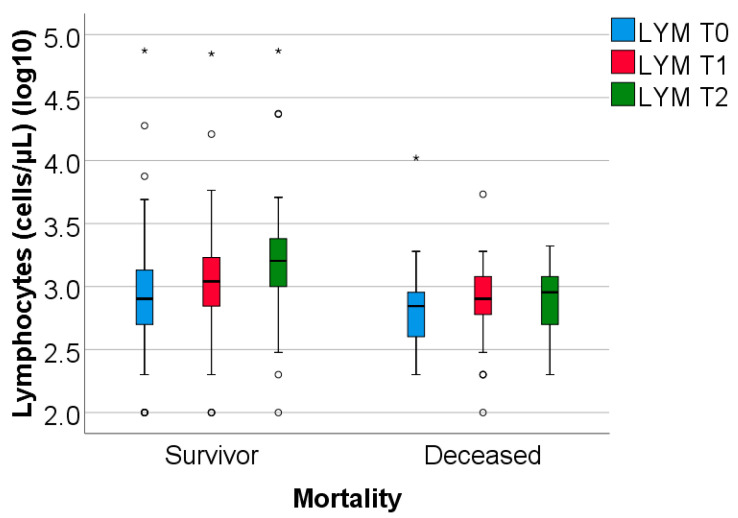
The dynamics of lymphocytes were observed at T0, T1, and T2. LYM = lymphocytes (illustrated as base10 logarithmic values). T0, T1, and T2 = Time 0, 1, and 2. (*) = outliers values.

The study group was divided based on outcome (deceased or discharged). Patients transferred to other hospitals, totaling 11, whose discharge status was unknown, were excluded from the study. The hematological parameters were evaluated by comparing their median values at T0, T1, and T2. The results are presented in Table 3 and Figure 2, Figure 3 and Figure 4.

Data from Table 3 and Figure 2, Figure 3 and Figure 4 show that while deceased patients had a stable inflammatory profile during admission, having no significant differences in evolution of leukocytes (*p* = 0.413), neutrophils (*p* = 0.468), lymphocytes (*p* = 0.190) or NLR (*p* = 0.852), surviving patients had a similar pattern to the general cohort, maintaining a significant decrease from T0 to T1/T2 of leukocytes/neutrophils/NLR (T0/T1: *p* < 0.001/*p* < 0.001/*p* < 0.001, T0/T2: *p* < 0.001/*p* < 0.001/*p* < 0.001), with no significant differences from T1 to T2 of leukocytes/neutrophils (*p* = 0.407/*p* = 0.056), NLR having a significant decrease from T1 to T2 (*p* < 0.001), associated with a significant increase in lymphocytes from T0 to T1 (*p* = 0.001), T0 to T2 (*p* < 0.001) and T1 to T2 (*p* < 0.001).

The difference in inflammatory profile evolution was confirmed by the comparison of T0 to T1 or T0 to T2 evolution differences in parameters between survival groups where the differences observed showed the same results: significant higher decreases in evolution of leukocytes/neutrophils/NLR in survivors vs. deceased (T0 to T1: *p* = 0.009/*p* = 0.002/*p* < 0.001, T0 to T2: *p* = 0.010/*p* = 0.006/*p* = 0.002) and significant higher increase in evolution of lymphocytes in survivors vs. deceased (T0 to T1: *p* = 0.023, T0 to T2: *p* < 0.001).

**Figure 5:**
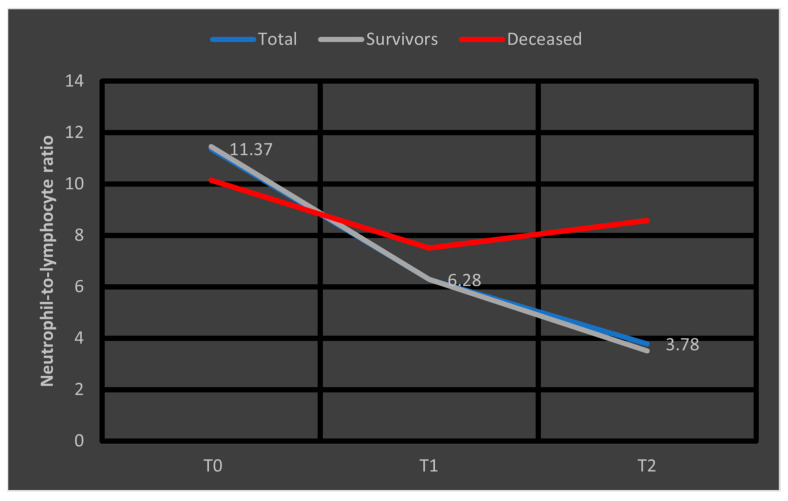
Median values of NLR at T0, T1, and T2. NLR = neutrophil-to-lymphocyte ratio. T0, T1, and T2 = Time 0, 1, and 2.

Regarding the dynamics of NLR values, we depicted in Figure 5 the median values at T0, T1, and T2 observed in the entire cohort, as well as separately in survivors and deceased patients. It is evident that, in survivors, these values approach the considered optimal range (1–3) [19], a trend not observed in patients who later died.

Below, we have highlighted the evolution of inflammatory parameters in the studied cohort over time, according to T0, T1, and T2 (Table 4):

Results from Table 4 show significant changes for all investigated parameters. Post hoc tests show a significant decrease in fibrinogen/CRP from T0 to T1 (*p* = 0.002/*p* < 0.001), T0 to T2 (*p* < 0.001/*p* < 0.001) and from T1 to T2 (*p* < 0.001/*p* < 0.001) while a significant decrease in procalcitonin was observed at T2 from T0 (*p* = 0.001) or T1 (*p* = 0.014), differences between T0 to T1 being not significant (*p* = 1.000). Comparison of inflammatory parameters between survival groups can be observed in Table 5.

Data from Table 5 show that deceased patients had no significant differences in fibrinogen evolution (*p* = 0.135) or procalcitonin (due to lack of valid cases), while CRP was significantly decreased only at T2 from T0 (*p* = 0.001), while differences between T0-T1 (*p* = 0.091) or T1-T2 (*p* = 0.470) were not significant. In survivors fibrinogen/CRP/procalcitonin were significantly decreased from T0 to T2 (*p* = 0.001/*p* < 0.001/*p* = 0.001) and from T1 to T2 (*p* < 0.001/*p* < 0.001/*p* = 0.010), while from T0 to T1 fibrinogen and CRP were significantly decreased (*p* = 0.009/*p* < 0.001) while procalcitonin had no significant differences (*p* = 1.000).

**Figure 6:**
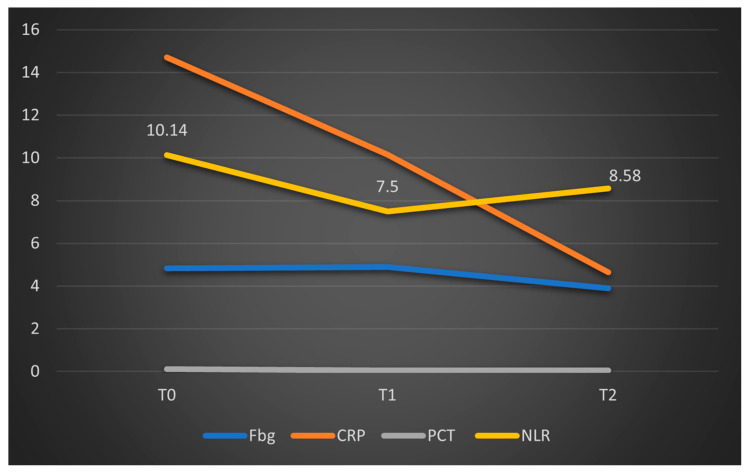
Dynamics of the median values of inflammatory parameters and NLR at T0, T1, and T2 in patients who died. Legend: Fbg = fibrinogen, CRP = C-reactive protein, PCT = procalcitonin, NLR = neutrophil-to-lymphocytes Ratio. T0, T1, and T2 = Time 0, 1, and 2.

**Figure 7:**
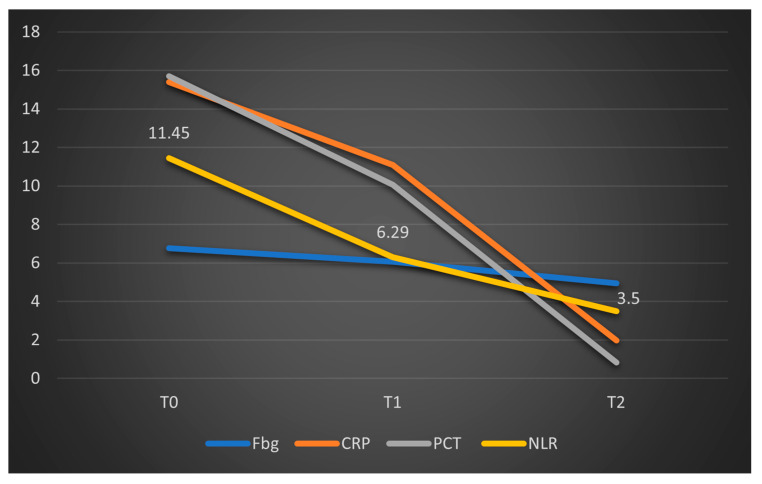
Dynamics of the median values of inflammatory parameters and NLR at T0, T1, and T2 in patients who survived. Legend: Fbg = Fibrinogen, CRP = C-reactive protein, PCT = procalcitonin, NLR = neutrophil-to-lymphocytes ratio. T0, T1, and T2 = Time 0, 1, and 2.

The limited number of valid data points analyzed at T1 and T2 in this group (deceased patients) may explain the lack of statistical significance for the parameters studied. However, the graphical comparison of median values, presented below, suggests a significant reduction in CRP levels, a trend that is not observed in fibrinogen, procalcitonin, or NLR (Figure 6). To facilitate comparison of the parameters, the obtained fibrinogen values were divided by 100. A similar representation was made for patients who survived in Figure 7, where CRP and procalcitonin had a significant reduction, along with NLR and fibrinogen had a stable evolution.

**Figure 8:**
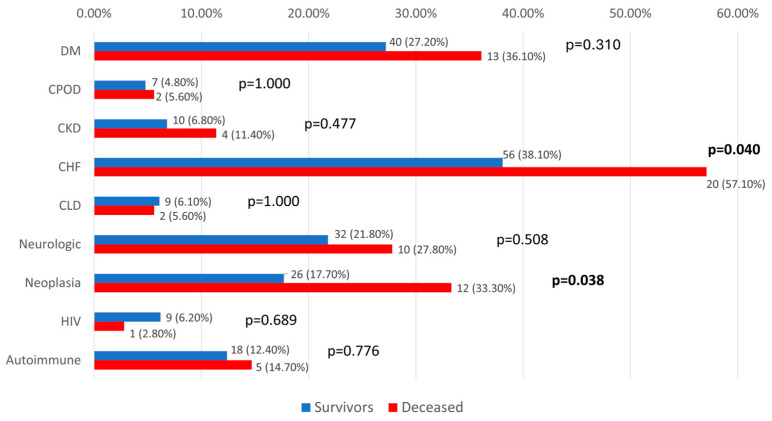
Distribution of analyzed patients according to comorbidities and mortality. DM = diabetes mellitus; CPOD = chronic obstructive pulmonary disease; CKD = chronic kidney disease; CHF = chronic heart failure; CLD = chronic liver disease; HIV = human immunodeficiency virus.

In our study, we assessed the potential implications related to the observed mortality. The results show that most of the tested comorbidities did not exhibit significant differences in relation to mortality (*p* > 0.05), except for chronic heart failure (57.1% vs. 38.1%, *p* = 0.040) and neoplasia (33.3% vs. 17.7%, *p* = 0.038), where patients with these comorbidities were significantly more associated with mortality (Figure 8).

Inflammatory parameters and NLR at T0 were compared in patients with CHF and neoplasia according to mortality in Table 6 and Table 7. Most of the parameters were not significantly different between survival groups for both comorbidities (*p* > 0.05), except for procalcitonin at T0 in patients with CHF, which was higher in survivors (median = 3.11, IQR = 0.56 to 17.7 vs. median = 0.98, IQR = 0.4–2.89, *p* = 0.046).

Differences in inflammatory parameters evolution pattern in patients with CHF or neoplasia were compared according to mortality in Table 8.

According to Table 8, in patients with CHF, fibrinogen/CRP/NLR were significantly lower at T2 versus T0 (*p* = 0.001/*p* < 0.001/*p* < 0.001)/T1 (*p* = 0.002/*p* < 0.001/*p* = 0.021) and at T1 versus T0 for CRP/NLR (*p* = 0.030/*p* = 0.021) while fibrinogen did not have a significant evolution from T0 to T1 (*p* = 0.269). Comparison between survival groups in patients with CHF shows that survivors have a similar pattern to the CHF group, having significantly lower fibrinogen/CRP/NLR at T2 versus T0 (*p* = 0.003/*p* < 0.001/*p* < 0.001)/T1 (*p* = 0.002 /*p* < 0.001/*p* = 0.005) and at T1 versus T0 for NLR (*p* = 0.012) while fibrinogen and CRP did not have a significant evolution from T0 to T1 (*p* = 1.000/*p* = 0.101). While deceased patients with CHF did not have a significant evolution of fibrinogen (*p* = 0.219) and NLR (*p* = 1.000), having only lower values of CRP at T2 versus T0 (*p* = 0.004), differences in CRP at T2 versus T1 (*p* = 0.438) or at T1 versus T0 (*p* = 0.221) were insignificant. Evolution differences comparisons between survival groups show significant differences only for NLR, where survivors had a significantly higher decrease in NLR from T0 to T1 (*p* = 0.019) or from T0 to T2 (*p* = 0.001) in comparison to deceased patients.

In patients with neoplasms, fibrinogen and CRP were significantly lower at T2 versus T0 (*p* = 0.006/*p* < 0.001)/T1 (*p* = 0.041/*p* = 0.049) and at T1 versus T0 for CRP (*p* = 0.049), while fibrinogen did not have a significant evolution from T0 to T1 (*p* = 0.286). NLR was not significantly different in evolution (*p* = 0.102). Comparison between survival groups in patients with neoplasms shows that survivors had significantly lower fibrinogen/CRP at T2 versus T0 (*p* = 0.046/*p* < 0.001) while differences from T0/T1 (*p* = 1.000/*p* = 0.065) or T1/T2 (*p* = 0.167/*p* = 0.065) were not significant. NLR was also not significantly different in evolution (*p* = 0.580). In deceased patients with neoplasms, none of the investigated parameters had a significant evolution (*p* > 0.05). Evolution differences comparisons between survival groups show insignificant differences (*p* > 0.05).

**Figure 9:**
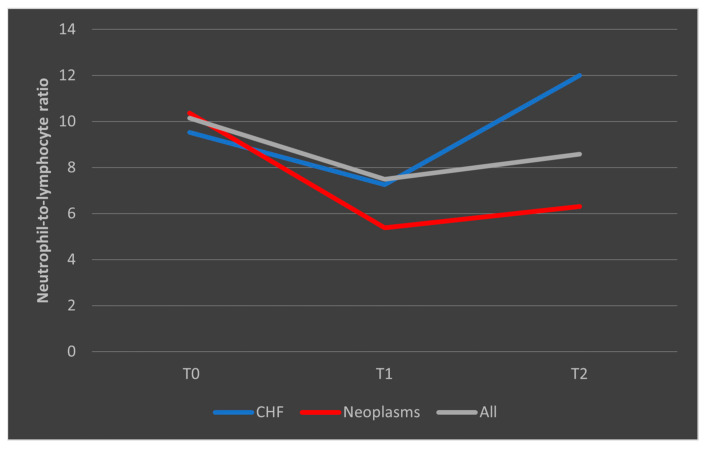
Evolution of NLR in patients who died, with known CHF or malignancies. CHF = cardiac heart failure. NLR = neutrophil-to-lymphocytes ratio. T0, T1, and T2 = Time 0, 1, and 2.

In the case of patients who died, we observed that among those with known congestive heart failure (CHF), the NLR values followed a decrease from T0 to T1, followed by a subsequent upward trend toward T2. In patients with malignancies, the trajectory was similar to the overall trend, with a higher decrease from T0 to T1 followed by a low increase from T1 to T2. However, all differences in NLR evolutions were not significant (*p* > 0.05) (Figure 9).

**Figure 10:**
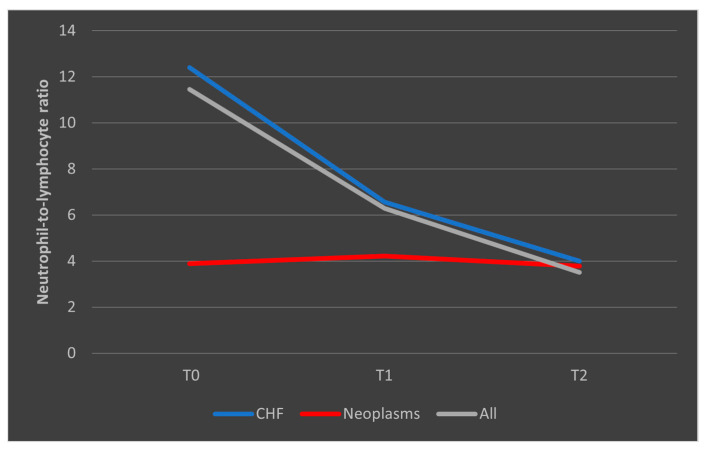
Evolution of NLR in patients who survived the sepsis episode. CHF = cardiac heart failure. NLR = neutrophil-to-lymphocytes ratio. T0, T1, and T2 = Time 0, 1, and 2.

In the case of surviving patients, we observed a decrease in the median NLR values among those with CHF, consistent with the overall trend seen in the entire group of survivors. Conversely, in patients with malignancies who survived the sepsis episode, NLR showed a steady and consistent evolution (Figure 10).

**Figure 11:**
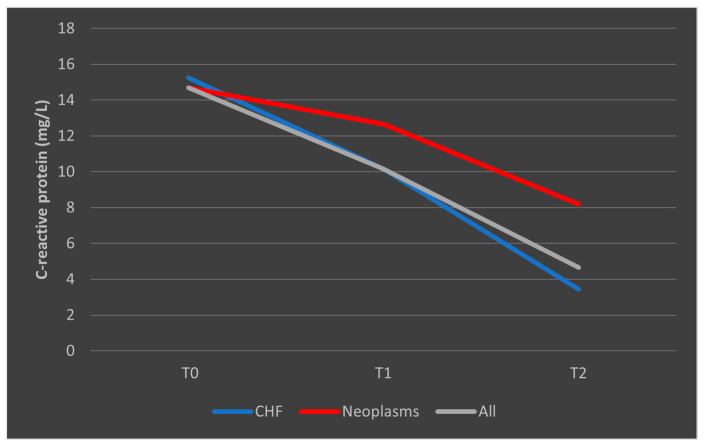
Evolution of CRP in patients with comorbidities who died. CHF = cardiac heart failure. CRP = C-reactive protein. T0, T1, and T2 = Time 0, 1, and 2.

**Figure 12:**
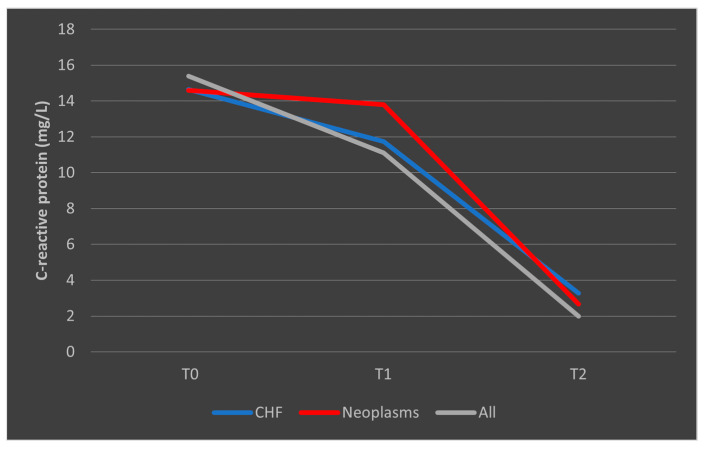
Evolution of CRP in patients with comorbidities who survived the sepsis episode. CHF = cardiac heart failure. CRP = C-reactive protein. T0, T1, and T2 = Time 0, 1, and 2.

In the case of CRP, deceased patients with CHF had a trajectory similar to the overall trend, while patients with malignancies had a consistent trajectory from T0 to T1 with a slight decrease from T1 to T2 (Figure 11). A similar trend was observed in patients who survived, as presented in Figure 12.

Inflammatory parameters and NLR were compared in patients with different age groups according to mortality in Table 9, Table 10 and Table 11. Most of the parameters were not significantly different between survival groups in all age groups (*p* > 0.05), except for fibrinogen at T0 in patients aged between 50 and 64 years, which was higher in survivors (759.4 ± 281 vs. 505.5 ± 211, *p* = 0.027).

In Table 12, we presented the median values of the inflammatory parameters at T0, T1, and T2, stratified by patient age groups. Since most deaths occurred in the age group over 65 years, patients were divided into two age categories.

Based on Table 12, in patients under 65 years of age, fibrinogen/CRP/NLR were significantly lower at T2 versus T0 (*p* < 0.001/*p* < 0.001/*p* < 0.001)/T1 (*p* = 0.001/*p* < 0.001/*p* = 0.008) and at T1 versus T0 for CRP (*p* = 0.025) while fibrinogen/NLR did not have a significant evolution from T0 to T1 (*p* = 0.247/*p* = 0.089). Comparison between survival groups in patients with age less than 65 years old shows that survivors have a similar pattern, having lower fibrinogen/CRP/NLR at T2 versus T0 (*p* = 0.003/*p* < 0.001/*p* < 0.001)/T1 (*p* = 0.004/*p* = 0.001/*p* = 0.003), and from T0 to T1 differences were not significant (*p* = 0.656/*p* = 0.075/*p* = 0.207). In deceased patients’ evolution of fibrinogen (*p* = 0.089) and NLR (*p* = 0.895) was not significant, while CRP was significantly lower only at T2 versus T0 (*p* = 0.048); differences between T0-T1 (*p* = 0.326) or T1-T2 (*p* = 1.000) were not significant. Evolution differences comparisons between survival groups show insignificant differences (*p* > 0.05). The reduction in fibrinogen levels at T2 to approximately 200 mg/dL is expected, considering the pathophysiological process of disseminated intravascular coagulation (DIC) and fibrinogen consumption, frequently observed in sepsis [20].

In patients with an age ≥ 65 years old, fibrinogen/CRP/NLR were significantly lower at T2 versus T0 (*p* < 0.001/*p* < 0.001/*p* < 0.001)/T1 (*p* = 0.001/*p* < 0.001/*p* = 0.002) and at T1 versus T0 (*p* = 0.011/*p* = 0.002/*p* < 0.001). Comparison between the survival groups in patients aged ≥ 65 years reveals a similar overall trend: levels of fibrinogen, CRP, and NLR were significantly lower at T2 compared to T0 (*p* < 0.001 for all), at T2 versus T1 (*p* = 0.001/*p* < 0.001/*p* = 0.002), and at T1 versus T0 (*p* = 0.019/*p* = 0.012/*p* < 0.001). In deceased patients’ evolution of fibrinogen (*p* = 0.558) and NLR (*p* = 0.829) was not significant, while CRP was significantly lower only at T2 versus T0 (*p* = 0.023); differences between T0–T1 (*p* = 0.407) or T1–T2 (*p* = 0.723) were not significant. Evolution differences comparisons between survival groups show significant differences only for NLR, where survivors had a significantly higher decrease in NLR from T0 to T1 (*p* < 0.001) or from T0 to T2 (*p* = 0.007) in comparison to deceased patients.

**Figure 13:**
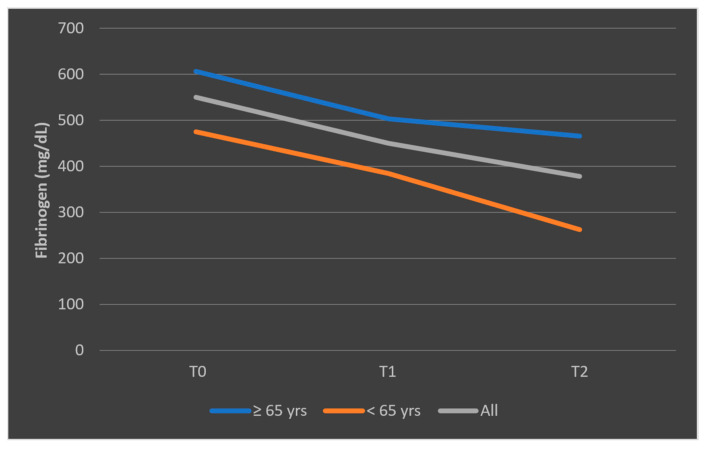
Evolution of mean fibrinogen levels in patients who died, according to age categories (<65 years, ≥65 years). T0, T1, and T2 = Time 0, 1, and 2.

**Figure 14:**
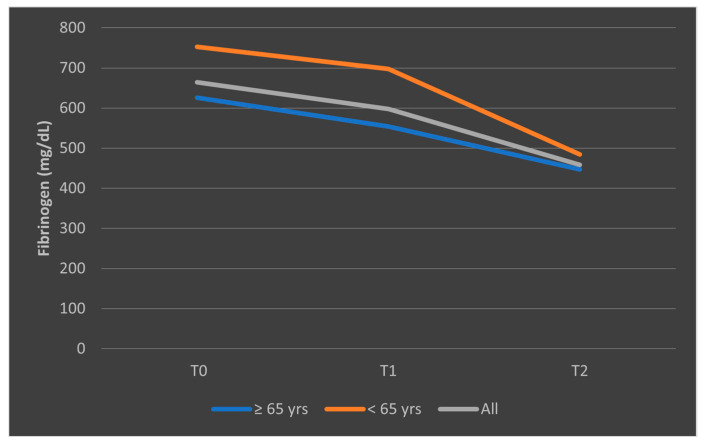
Evolution of mean fibrinogen levels in patients who survived the sepsis episode, according to age categories (<65 years, ≥65 years). T0, T1, and T2 = Time 0, 1, and 2.

The different pattern observed in the mean fibrinogen levels among patients who died, stratified by age categories, is presented in Figure 13. The representation of mean fibrinogen evolution was also presented in Figure 14 for patients who survived the sepsis episode.

## 4. Discussion

Our study evaluated a cohort of 198 patients hospitalized in 2024 with a diagnosis of sepsis at a tertiary hospital specializing in infectious diseases. The cohort demonstrated a relatively balanced gender distribution, with a higher proportion of patients over 65 years old (62.3%), predominantly from urban areas (81.9%). Most patients (51.5%) had experienced more than two hospitalizations in the preceding 12 months.

Medical history revealed that 41.9% had chronic cardiac conditions, 28.1% had diabetes mellitus, 20.6% had malignancies, and 28.6% were documented to have immunodeficiency. The primary sources of sepsis were pulmonary (30.1%), urinary (21%), and gastrointestinal (19.9%) infections, findings consistent with previous reports indicating pulmonary, urinary, and intra-abdominal infections as leading sources of sepsis [1,8].

Pathogen identification was achieved in 61.3% of cases, with Gram-negative bacteria being most prevalent, followed by Gram-positive cocci. Overall, 75.8% of patients were discharged, while 18% succumbed to the disease.

We analyzed the evolution of White Blood Cell (WBC) counts and cell subsets (neutrophils and lymphocytes) at T0, T1, and T2. Significant decreases were observed between T0 and T1 across all parameters, which persisted between T0 and T2. Only survivors exhibited a continuous decline in leukocytes and neutrophils from T0 to T1 and T2. Conversely, lymphocyte counts increased over time in survivors, whereas in non-survivors, the increase was absent or minimal. This pattern is illustrated in Figure 5, where at T2, the median NLR decreased to approximately 4 in survivors, while in non-survivors, despite a lower initial median, NLR increased one week after admission. This trend suggests that rising NLR after one week may serve as a prognostic indicator of adverse outcomes in septic patients.

Multiple studies [17,21,22] have similarly identified NLR as a significant predictor of mortality in sepsis and septic shock. Our findings support the hypothesis that an ascending NLR trend after seven days of therapy could signify a worse prognosis.

Regarding inflammatory markers (fibrinogen, CRP, and PCT), we observed a statistically significant decrease from T0 to T1, and correspondingly from T0 to T2 within the overall cohort. When stratified into survivors and non-survivors, this pattern persisted among survivors, with significant differences between T0 and T2. In the deceased subgroup, only CRP showed a significant decrease (*p* < 0.01), whereas fibrinogen levels did not change significantly (*p* = 0.135), and similar non-significant trends were observed for NLR (*p* = 0.853). These results are summarized in Figure 6.

Since comorbidity analysis revealed that heart failure and malignancies were more frequently associated with mortality, we examined the evolution of inflammatory parameters and NLR according to outcome (survivors vs. non-survivors). No significant differences were observed at T0 (see Table 6 and Table 7). Among patients with CHF or malignancies who died, NLR did not have a significant evolution. Survivors within these subgroups showed a decrease in values over time, similar to the overall survivor group, with NLR around 4 at T2.

Therefore, we concluded that NLR measured at one week of hospitalization can be a predictive factor for mortality. Patients with NLR ≥ 8 could have a high likelihood of death, whereas those with NLR ≤4 are possibly more likely to survive sepsis. This observation excludes the subgroup of patients with malignancies, where NLR behavior was different; in this group, the median values evolved similarly in both survivors and non-survivors.

Further analysis of fibrinogen and CRP from T0 to T2 in patients with CHF and malignancies, compared to the entire cohort, revealed no significant overall differences, values had a similar pattern to the overall tendency in survivors’ groups, with mostly no significant differences in the mortality group (except for CRP).

Stratifying by age groups (<65 vs. ≥65 years) did not reveal statistically significant differences at T0 concerning NLR or inflammatory parameters in both age groups. In survivors’ inflammatory parameters and NLR had a significant decrease, while in the mortality group, only CRP was significantly different.

In this study involving 198 patients, we aimed to identify potential predictors of unfavorable evolution after 2–3 days of in-hospital progression (the immediate consequence of initial empiric therapy at admission) or at 7–8 days (the result of more targeted antimicrobial treatments). In our cohort, no clear predictive pattern was observed within the first 48–72 h. However, our findings demonstrate that certain hematological and inflammatory parameters measured one week after admission can serve as reliable predictors of adverse outcomes.

Several additional biomarkers have been proposed for sepsis prognostication, including serum lactate, endothelin-1, angiopoietin-2, and syndecan-1 [23,24,25,26]. Nevertheless, we believe that the temporal evolution of easily monitored parameters evaluated in this study can serve as a useful predictor of mortality risk in this patient population.

The present study has several clear limitations: its retrospective design, single-center setting, and the relatively short study period, which yielded limited data within the analyzed subgroups. Consequently, a prospective study with a larger sample size would likely provide stronger statistical support for our conclusions.

## 5. Conclusions

The study conducted on patients with sepsis admitted in 2024 at a tertiary hospital specialized in infectious diseases found that, at seven days after hospital admission, a non-significant decrease or, more notably, an increase in the neutrophil/lymphocyte ratio (NLR), or a non-significant decrease in fibrinogen levels—especially in patients over 65 years old—could serve as predictive factors for an unfavorable outcome. Conversely, at the same point in time, an NLR around 4, or less, along with a statistically significant and consistent decrease in fibrinogen, CRP, or procalcitonin, could represent elements that predict patient survival after a sepsis episode.

## Figures and Tables

**Table 1:** Characteristics of the analyzed patients.

Parameter		Value
***Number of cases, %***		198 (100%)
***Gender (Female) (Nr., %)***		101 (50.8%)
***Age (Mean*** *± **SD) (Median (IQR))***		65.05 ± 17.23, 69 (56–77)
***Age group (Nr., %)***		
**<50 years**		31 (15.6%)
**50–64 years**		44 (22.1%)
**≥65 years**		124 (62.3%)
***Environment (Urban) (Nr., %)***		163 (81.9%)
***Institutionalized (Nr., %) (N =* 198*)***		13 (6.6%)
***Multihospitalized (Nr., %) (N =* 198*)***		102 (51.5%)
***Medical history (Nr., %) (Total)***		
**Diabetes mellitus**		56 (28.1%)
**CPOD**		11 (5.5%)
**CKD (*N =* 198)**		15 (7.6%)
**CHF (*N =* 198)**		83 (41.9%)
**CLD**		13 (6.5%)
**Neurological diseases**		45 (22.6%)
**Neoplasia**		41 (20.6%)
**HIV (*N =* 198)**		11 (5.6%)
**Autoimmune diseases (*N =* 195)**		27 (13.8%)
***Immunosuppression status***		
**Unknown**		142 (71.4%)
**Present**		57 (28.6%)
***Infection starting point (Nr., %) (N =* 186*)***		
**Cutaneous**		30 (16.2%)
**Digestive**		37 (19.9%)
**Mixed**		22 (11.8%)
**Osseous**		2 (1.1%)
**Respiratory**		56 (30.1%)
**Urinary**		39 (21%)
***Type of infection (Nr., %) (Total)***		
**Identified microorganism**		122 (61.3%)
**Gram-positive cocci (*N =* 185)**		26 (14.1%)
**Gram-negative cocci (*N =* 185)**		0 (0%)
**Gram-positive bacilli (*N =* 185)**		18 (9.7%)
**Gram-negative bacilli (*N =* 185)**		86 (46.5%)
**Fungi (*N =* 178)**		14 (7.9%)
**Mycobacterium tuberculosis (*N =* 125)**		1 (0.8%)
***Antibiotic resistance (Nr., %) (N =* 143*)***		41 (28.7%)
***Type of resistance (Nr., %) (N =* 39*) (Total)***		
**Gram-negative bacilli**	**NDM + OXA-48**	13 (33.3%)
	**NDM**	17 (43.58%)
	**OXA-48**	17 (43.58%)
	**ESBL**	10 (25.64%)
	**OXA-23**	3 (7.69%)
	**KPC**	3 (7.69%)
**Gram-positive cocci**	**VRE**	3 (7.69%)
	**MRSA**	3 (7.69%)
	**High-grade penicillinase**	2 (5.12%)
***T*0*—Antibiotic treatment (Total)***		
**Aminoglycosides**		7 (3.5%)
**Beta-lactamase inhibitors**		14 (7%)
**Carbapenems**		105 (52.8%)
**Cephalosporins**		63 (31.7%)
**Cyclins**		25 (12.6%)
**Fluoroquinolones**		13 (6.5%)
**Glycopeptides**		83 (41.7%)
**Lincosamides**		1 (0.5%)
**Monobactams**		5 (2.5%)
**Nitroimidazols**		7 (3.5%)
**Oxazolidinones**		13 (6.5%)
**Penicilins**		8 (4%)
**TMP/SMX**		8 (4%)
***Antibiotic treatment duration (days) (Mean*** ± ***SD)*** ***(Median (IQR)) (N =* 173*)***		8.72 ± 6.16, 7 (4–12)
***Antifungal treatment (Nr., %) (N =* 193*)***		39 (20.2%)
***Anti-TB agents (N =* 164*) (Nr., %)***		4 (2.4%)
***Corticosteroid therapy (Nr., %) (N =* 193*)***		41 (21.2%)
***Corticosteroid therapy duration (days) (Mean*** ± ***SD)*** ***(Median (IQR)) (N =* 34*)***		9.15 ± 5.91, 7 (6.75–10)
***Hospitalization duration (Mean*** ± ***SD) (Median (IQR)) (N =* 186*)***		14.7 ± 10.39, 12 (8–18)
***ICU admission (Nr., %) (N =* 192*)***		26 (13.5%)
***Discharge status (Nr., %) (N =* 194*)***		
**Discharged**		147 (75.8%)
**Transferred**		11 (5.7%)
**Deceased**		36 (18.6%)
***Laboratory parameters (Mean*** ± ***SD) (Median (IQR))—T*0**		
**Leukocytes (*N =* 195) (cells/μL**)		13,659.49 ± 11,107.88, 11,900 (7900–16,700)
**Neutrophils (*N =* 194) (cells/μL**)		11,288.14 ± 9011.53, 9500 (6300–14,525)
**Lymphocytes (*N =* 194) (cells/μL**)		1548.45 ± 5545.27, 800 (500–1325)
**Fibrinogen (*N =* 164) (mg/dL)**		622.34 ± 226.39, 605 (459.25–746.50)
**CRP (*N =* 193) (mg/L)**		16.55 ± 10.97, 15.3 (8.15–24.3)
**PCT (*N =* 124) (ng/mL)**		9.36 ± 16.3, 2.26 (0.51–9.95)

SD = standard deviation, IQR = interquartile range, CPOD = chronic obstructive pulmonary disease, CKD = chronic kidney diseases, CHF = chronic heart failure, CLD = chronic liver disease, HIV = human immunodeficiency virus, NDM = New Delhi metallo-β-lactamase, OXA = oxacillinase, ESBL = extended-spectrum beta-lactamase, KPC = Klebsiella pneumoniae carbapenemase, VRE = Vancomycin-Resistant Enterococci, MRSA = Methicillin-Resistant Staphylococcus Aureus, T0 = Time 0, TMP/SMX = trimethoprim/sulfamethoxazole, TB = tuberculosis, ICU = intensive care unit, CRP = C-reactive protein, PCT = procalcitonin.

**Table 2:** Evolution comparison of WBCs and subsets parameters.

Parameter (Median (IQR))	T0	T1	T2	*p*
**Leukocytes (cells/μL)**	12,350 (7975–16,625)	9250 (6300–14,800)	9150 (6300–13,700)	<0.001 *
**Neutrophils (cells/μL)**	9800 (6550–14650)	7100 (4150–12,000)	6300 (3900–9800)	<0.001 *
**Lymphocytes (cells/μL)**	800 (450–1300)	1100 (700–1600)	1500 (900–2100)	<0.001 *
**NLR**	11.37 (5.75–22.72)	6.28 (3.1–12.85)	3.78 (2.38–7.12)	<0.001 *

* Related-samples Friedman’s two-way analysis of variance by ranks; T0, 1, and 2 = Time 0, 1, and 2; IQR = interquartile range; NLR = neutrophil-to-lymphocyte ratio.

**Table 3:** Evolution comparison of WBCs and subsets parameters according to mortality.

Parameter (Median (IQR))		T0	T1	T2	*p* *
**Leukocytes (cells/μL**)	**Deceased** **(*N =* 26)**	9350 (6175–15,050)	9150 (5200–14,925)	9800 (7000–14,725)	0.413
	**Survivors** **(*N =* 124)**	12,900 (8300–17,000)	9200 (6450–14,800)	9100 (6025–13,200)	**<0.001**
	*p* **	**0.009/0.010**			-
**Neutrophils (cells/μL**)	**Deceased** **(*N =* 25)**	7500 (4150–13,100)	7000 (3900–12,800)	7100 (4750–11,600)	0.468
	**Survivors** **(*N =* 124)**	10,400 (6825–14,775)	7100 (4125–12,600)	5850 (3825–9475)	**<0.001**
	*p* **	**0.002/0.006**			-
**Lymphocytes (cells/μL**)	**Deceased** **(*N =* 25)**	700 (400–950)	800 (550–1250)	900 (500–1400)	0.190
	**Survivors** **(*N =* 124)**	800 (500–1375)	1100 (700–1700)	1600 (1000–2400)	**<0.001**
	*p* **	**0.023/<0.001**			-
**NLR**	**Deceased (*N =* 25)**	10.14 (3.51–20)	7.5 (3.51–17.97)	8.58 (4.6–17.31)	0.852
	**Survivors (*N =* 124)**	11.45 (5.96–23.27)	6.29 (3.05–12.26)	3.5 (2.21–5.86)	**<0.001**
	*p* **	**<0.001/0.002**			-

* Related-samples Friedman’s two-way analysis of variance by ranks, ** Mann–Whitney U test (T0 vs. T1 difference/T0 vs. T2 difference between survival groups); T0, 1, and 2 = Time 0, 1, and 2; IQR = interquartile range; NLR = neutrophil-to-lymphocyte ratio.

**Table 4:** Evolution comparison of inflammatory parameters.

Parameter (Median (IQR))	T0	T1	T2	*p*
**Fibrinogen (Mean ± SD) (mg/dL)**	649.92 ± 251.7	581.92 ± 235.87	451 ± 173.88	<0.001 **
**CRP (mg/L)**	15.5 (8.04–24.4)	11.3 (4.71–17.4)	2.39 (0.89–6.37)	<0.001 *
**Procalcitonin (ng/mL)**	14.45 (2.09–45.44)	8.72 (2.79–30.59)	0.69 (0.24–1.32)	0.001 *

* Related-samples Friedman’s two-way analysis of variance by ranks, ** Repeated-Measures One-Way ANOVA with Greenhouse–Geisser Correction, SD = Standard deviation, IQR = interquartile range; T0, 1, and 2 = Time 0, 1, and 2; CRP = C-reactive protein.

**Table 5:** Evolution comparison of inflammatory parameters according to mortality.

Parameter (Median (IQR))		T0	T1	T2	*p* *
**Fibrinogen** **(Mean** ± **SD) (mg/dL)**	**Deceased** **(*N =* 7)**	550.86 ± 215.63	452.43 ± 112.48	379.43 ± 190.53	0.135 ***
	**Survivors** **(*N =* 43)**	664.28 ± 256.27	597.47 ± 241.11	458.05 ± 169.61	<0.001 ***
	*p* **	0.240/0.568			-
**CRP (mg/L)**	**Deceased** **(*N =* 18)**	14.7 (12.03–17.93)	10.15 (1.9–16.18)	4.65 (2.17–10.63)	**0.001**
	**Survivors** **(*N =* 94)**	15.4 (6.92–24.85)	11.1 (5.09–17.42)	1.98 (0.81–5.65)	**<0.001**
	*p* **	0.444/0.189			-
**Procalcitonin (ng/mL)**	**Deceased (*N =* 1)**	0.11	0.04	0.04	-
	**Survivors (*N =* 13)**	15.7 (3.79–47.66)	10.06 (4.75–31.54)	0.83 (0.28–1.4)	**0.001**
	*p* **	**-**			-

* Related-samples Friedman’s two-way analysis of variance by ranks, ** Mann–Whitney U Test (T0 vs. T1 difference/T0 vs. T2 difference between survival groups), *** Repeated-Measures One-Way ANOVA; SD = standard deviation; IQR = interquartile range; T0, 1, and 2 = Time 0, 1, and 2; CRP = C-reactive protein.

**Table 6:** Comparison of inflammatory parameters and NLR at T0 in patients with CHF according to mortality.

Fibrinogen T0 (mg/dL)	Mean ± SD	Median (IQR)	Mean Rank	*p* ***
Survivor (*p* = 0.798 **)	623.5 ± 210	607 (493.5–770.5)	-	0.912
Deceased (*p* = 0.496 **)	617 ± 156.6	634 (482–747)	-	
**CRP T0 (mg/L)**	**Mean** ± **SD**	**Median (IQR)**	**Mean Rank**	*p* *
Survivor (*p* = 0.025 **)	15.06 ± 10	13.85 (7.1–21.4)	38.47	0.986
Deceased (*p* = 0.063 **)	13.9 ± 5.7	15.6 (11.4–17.3)	38.58	
**Procalcitonin T0 (ng/mL)**	**Mean** ± **SD**	**Median (IQR)**	**Mean Rank**	*p* *
Survivor (*p* < 0.001 **)	11.6 ± 17.9	3.11 (0.56–17.7)	26.70	0.046
Deceased (*p* = 0.017 **)	1.3 ± 1.2	0.98 (0.4–2.89)	17.09	
**NLR T0**	**Mean** ± **SD**	**Median (IQR)**	**Mean Rank**	*p* *
Survivor (*p* < 0.001 **)	20 ± 21.5	12.6 (6.5–23.1)	40.04	0.163
Deceased (*p* < 0.001 **)	12.7 ± 13	8.7 (5.1–14.5)	31.97	

* Mann–Whitney U Test; ** Shapiro–Wilk Test; *** Student *t*-Test; SD = standard deviation, IQR = interquartile range; T0 = Time 0; CRP = C-reactive protein; NLR = neutrophil-to-lymphocyte ratio.

**Table 7:** Comparison of inflammatory parameters and NLR at T0 in patients with neoplasia according to mortality.

Fibrinogen T0 (mg/dL)	Mean ± SD	Median (IQR)	Mean Rank	*p* ***
Survivor (*p* = 0.796 **)	594 ± 225	633 (404–728.5)	-	0.655
Deceased (*p* = 0.310 **)	555 ± 196	610 (385–726)	-	
**CRP T0 (mg/L)**	**Mean** ± **SD**	**Median (IQR)**	**Mean Rank**	*p* *
Survivor (*p* = 0.203 **)	18.24 ± 13.3	16.9 (8.9–27.2)	19.96	0.421
Deceased (*p* < 0.001 **)	15.7 ± 13.3	14.6 (7.4–15.9)	16.73	
**Procalcitonin T0 (ng/mL)**	**Mean** ± **SD**	**Median (IQR)**	**Mean Rank**	*p* *
Survivor (*p* < 0.001 **)	6.33 ± 10.6	1.16 (0.34–6.78)	15.22	0.555
Deceased (*p* < 0.001 **)	5.2 ± 12.7	0.79 (0.33–2.97)	13.20	
**NLR T0**	**Mean** ± **SD**	**Median (IQR)**	**Mean Rank**	*p* *
Survivor (*p* < 0.001 **)	13.4 ± 20	4.68 (1.8–15.87)	19.31	0.889
Deceased (*p* < 0.001 **)	9.53 ± 12.1	6.81 (2.04–11.5)	19.92	

* Mann–Whitney U Test; ** Shapiro–Wilk Test; *** Student *t*-Test; SD = standard deviation; IQR = interquartile range; T0 = Time 0; CRP = C-reactive protein; NLR = neutrophil-to-lymphocyte ratio.

**Table 8:** Evolution comparison of fibrinogen, CRP, and NLR according to mortality in patients with CHF or neoplasia.

Parameter (Median (IQR))			T0	T1	T2	*p* *
**CHF**	**Fibrinogen** **(Mean** ± **SD) (mg/dL)**	**All (*N =* 24)**	611.7 ± 241.62	571.8 ± 246.34	430.1 ± 188.22	<0.001 ***
		**Deceased (*N =* 6)**	593.3 ± 201.6	482.5 ± 87.1	414.3 ± 182.56	0.219 ***
		**Survivors (*N =* 17)**	607.6 ± 262.8	590.3 ± 280.1	424.6 ± 195.33	<0.001 ***
		*p* **	0.121/1.000			**-**
	**CRP (mg/L)**	**All (*N =* 51)**	14.8 (8.47–19.7)	11.6 (6.51–16.4)	3.39 (0.98–8.28)	**<0.001**
		**Deceased (*N =* 10)**	15.25 (12.03–16.53)	10.15 (2–15.43)	3.43 (2.03–8.65)	**0.004**
		**Survivors (*N =* 40)**	14.65 (7.09–24)	11.75 (6.63–17.05)	3.28 (0.91–7.8)	**<0.001**
		*p* **	0.758/0.462			**-**
	**NLR**	**All (*N =* 66)**	11.6 (6.4–22.48)	6.45 (3.45–13.5)	4.22 (2.62–8.47)	**<0.001**
		**Deceased (*N =* 14)**	9.52 (3.61–14.61)	7.26 (3.7–17.08)	12 (2.9–18.38)	1.000
		**Survivors (*N =* 51)**	12.41 (6.42–23.3)	6.57 (3.16–13.33)	4 (2.61–5.92)	**<0.001**
		*p* **	**0.019/0.001**			**-**
**Neoplasms**	**Fibrinogen** **(Mean** ± **SD) (mg/dL)**	**All (*N =* 12)**	**614.8** ± 188.76	**572.8** ± 207.06	**438.3** ± 135.2	0.002 ***
		**Deceased (*N =* 2)**	674.5 ± 91.21	561 ± 14.14	304.5 ± 119.5	-
		**Survivors (*N =* 9)**	632.4 ± 192.52	608.3 ± 215.71	486.9 ± 112.41	0.024 ***
		*p* **	0.267/0.225			**-**
	**CRP (mg/L)**	**All (*N =* 25)**	**14.8** **(10.25–24.3)**	**13.9** **(5.71–19.25)**	**3.5** **(1.58–11.15)**	<0.001
		**Deceased** **(*N =* 8)**	14.7 (12.45–17.7)	12.65 **(5.89–19.42)**	8.2 (3.39–14.98)	0.325
		**Survivors** **(*N =* 16)**	14.6 (6.09–26.85)	13.8 (3.93–19.33)	2.67 (0.89–7.81)	**<0.001**
		*p* **	0.155/0.457			**-**
	**NLR**	**All (*N =* 33)**	5.48 (1.9–15.59)	4.5 (2.85–10.94)	4.5 (1.66–11.5)	0.102
		**Deceased** **(*N =* 8)**	10.37 (3.09–12.21)	5.39 (3.31–10.54)	6.3 (3.45–10.97)	0.197
		**Survivors** **(*N =* 23)**	3.88 (1.79–18.85)	4.21 (2.42–12.23)	3.78 (1.35–14.4)	0.580
		*p* **	-			**-**

* Related-samples Friedman’s Two-Way analysis of variance by ranks; ** Mann–Whitney U Test (T0 vs. T1 difference/T0 vs. T2 difference between survival groups); *** Repeated-Measures One-Way ANOVA with Greenhouse–Geisser correction; SD = standard deviation; IQR = interquartile range; T0, 1, and 2 = Time 0, 1, and 2; CRP = C-reactive protein; NLR = neutrophil-to-lymphocyte ratio.

**Table 9:** Comparison of inflammatory parameters and NLR at T0 in patients < 50 years old with CHF according to mortality.

Fibrinogen T0 (mg/dL)	Mean ± SD	Median (IQR)	Mean Rank	*p* *
Survivor (*p* = 0.830 **)	594 ± 219	604 (485–728)	10.00	1.000
Deceased (*p=* -**)	610.5 ± 181	610.5 (482–739)	10.00	
**CRP T0 (mg/L)**	**Mean** ± **SD**	**Median (IQR)**	**Mean Rank**	*p* *
Survivor (*p* = 0.095 **)	18.58 ± 10.7	19.9 (10.6–27.6)	13.92	0.394
Deceased (*p=* -**)	10.91 ± 7.06	10.91 (5.9–15.9)	8.50	
**Procalcitonin T0 (ng/mL)**	**Mean** ± **SD**	**Median (IQR)**	**Mean Rank**	*p* *
Survivor (*p* = 0.002 **)	10.76 ± 13.1	6.24 (3.4–10.1)	6.70	0.758
Deceased (*p=* -**)	10.7 ± 15	10.77 (0.1–21.4)	5.50	
**NLR T0**	**Mean** ± **SD**	**Median (IQR)**	**Mean Rank**	*p* *
Survivor (*p* = 0.001 **)	25 ± 21.93	17.4 (9–34.9)	13.62	0.812
Deceased (*p=* -**)	15.3 ± 6.64	15.3 (10.6–20)	12.00	

* Mann–Whitney U Test; ** Shapiro–Wilk Test; SD = standard deviation; IQR = interquartile range; T0 = Time 0; CRP = C-reactive protein; NLR = neutrophil-to-lymphocyte ratio.

**Table 10:** Comparison of inflammatory parameters and NLR at T0 in patients aged between 50 and 64 years with neoplasia according to mortality.

Fibrinogen T0 (mg/dL)	Mean ± SD	Median (IQR)	Mean Rank	*p* ***
Survivor (*p* = 0.221 **)	759.4 ± 281	794 (489–1076)	-	0.027
Deceased (*p* = 0.358 **)	505.5 ± 211	457.5 (321–741.5)	-	
**CRP T0 (mg/L)**	**Mean** ± **SD**	**Median (IQR)**	**Mean Rank**	*p* *
Survivor (*p* = 0.055 **)	19.7 ± 12.7	20.2 (6.1–31.9)	21.97	0.343
Deceased (*p* = 0.006 **)	17 ± 14.9	15 (8–18.4)	17.56	
**Procalcitonin T0 (ng/mL)**	**Mean** ± **SD**	**Median (IQR)**	**Mean Rank**	*p* *
Survivor (*p* < 0.001 **)	8.44 ± 15.6	1.93 (0.51–10.6)	15.86	0.354
Deceased (*p* = 0.007 **)	1.5 ± 1.3	0.86 (0.6–3.2)	12.29	
**NLR T0**	**Mean** ± **SD**	**Median (IQR)**	**Mean Rank**	*p* *
Survivor (*p* = 0.349 **)	7.94 ± 4.6	7.85 (3.88–11.85)	22.32	0.414
Deceased (*p* = 0.031 **)	8.5 ± 9.8	4.88 (1.49–14.37)	18.50	

* Mann–Whitney U Test; ** Shapiro–Wilk Test; *** Student *t*-Test; SD = standard deviation; IQR = interquartile range; T0 = Time 0; CRP = C-reactive protein; NLR = neutrophil-to-lymphocyte ratio.

**Table 11:** Comparison of inflammatory parameters and NLR at T0 in patients ≥ 65 years old with CHF according to mortality.

Fibrinogen T0 (mg/dL)	Mean ± SD	Median (IQR)	Mean Rank	*p* *
Survivor (*p* = 0.063 **)	615.7 ± 202	601 (484–727)	53.24	0.374
Deceased (*p* = 0.014 **)	591 ± 205	538 (437–675)	46.50	
**CRP T0 (mg/L)**	**Mean** ± **SD**	**Median (IQR)**	**Mean Rank**	*p* *
Survivor (*p* < 0.001 **)	15.54 ± 10.5	13.85 (7.5–22.2)	58.29	0.622
Deceased (*p* = 0.014**)	13.8 ± 7.9	13.75 (8.5–17.3)	54.54	
**Procalcitonin T0 (ng/mL)**	**Mean** ± **SD**	**Median (IQR)**	**Mean Rank**	*p* *
Survivor (*p* < 0.001 **)	11.2 ± 17.8	2.36 (0.44–15.68)	37.69	0.598
Deceased (*p* < 0.001 **)	10.3 ± 21	1.84 (0.44–4.93)	34.53	
**NLR T0**	**Mean** ± **SD**	**Median (IQR)**	**Mean Rank**	*p* *
Survivor (*p* < 0.001 **)	21.1 ± 22.5	12.4 (5.9–25.7)	59.79	0.151
Deceased (*p* < 0.001 **)	14.6 ± 15.9	7.87 (4.7–14.7)	48.90	

* Mann–Whitney U Test; ** Shapiro–Wilk Test; SD = standard deviation; IQR = interquartile range; T0 = Time 0; CRP = C-reactive protein; NLR = neutrophil-to-lymphocyte ratio.

**Table 12:** Evolution comparison of fibrinogen, CRP, and NLR according to mortality in patients stratified by age.

Parameter (Median (IQR))			T0	T1	T2	*p* *
**<65 years**	**Fibrinogen** **(Mean** ± **SD) (mg/dL)**	**All (*N =* 18)**	686.06 ± 328.12	627.11 ± 304.21	443 ± 198.29	<0.001 ***
		**Deceased (*N =* 3)**	477 ± 232.34	385.33 ± 146.67	263.33 ± 113.02	0.094 ***
		**Survivors (*N =* 13)**	753.08 ± 341.56	697.08 ± 308.37	484.31 ± 196.34	<0.001 ***
		*p* **	0.148/1.000			-
	**CRP (mg/L)**	**All (*N =* 43)**	19.2 (5.25–28.1)	15.8 (4.91–22.1)	2.54 (1.29–8.09)	**<0.001**
		**Deceased (*N =* 7)**	14.6 (3.23–18.5)	7.59 (1.56–18)	3.35 (2.09–12.2)	**0.049**
		**Survivors (*N =* 34)**	20.5 (5.12–28.53)	15.75 (5.87–22.53)	2.45 (0.97–7.66)	**<0.001**
		*p* **	0.550/0.253			-
	**NLR**	**All (*N =* 61)**	8.95 (4.78–16.33)	5.83 (3.07–10.9)	3.78 (2.14–6.75)	**<0.001**
		**Deceased (*N =* 9)**	4.88 (1.49–20)	4 (1.53–14.03)	7.88 (5.5–13.98)	0.895
		**Survivors (*N =* 49)**	9.18 (5.86–15.27)	6.84 (3.38–11.92)	3.25 (2.05–5.36)	**<0.001**
		*p* **	0.208/0.106			-
**≥65 years**	**Fibrinogen** **(Mean** ± **SD) (mg/dL)**	**All (*N =* 35)**	631.3 ± 204.97	558.7 ± 192.82	455.2 ± 162.87	<0.001 ***
		**Deceased (*N =* 4)**	606.2 ± 217.86	502.7 ± 55.52	466.5 ± 201.25	0.558 ***
		**Survivors (*N =* 30)**	625.8 ± 204.39	554.3 ± 196.13	446.6 ± 158.97	<0.001 ***
		*p* **	0.868/0.449			-
	**CRP (mg/L)**	**All (*N =* 72)**	14.55 (8.15–19.5)	9 (4.65–15.7)	1.96 (0.85–5.77)	**<0.001**
		**Deceased (*N =* 11)**	14.8 (12.3–17.8)	11.4 (2.1–16.1)	5.79 (2.39–10.1)	**0.026**
		**Survivors (*N =* 60)**	14.4 (7.09–21.43)	9 (4.82–15.7)	1.82 (0.76–5.23)	**<0.001**
		*p* **	0.567/0.528			-
	**NLR**	**All (*N =* 92)**	12.16 (6.08–28.83)	6.29 (3.13–12.96)	3.85 (2.52–7.77)	**<0.001**
		**Deceased (*N =* 16)**	11 (6.37–25.7)	9.4 (4.33–18.71)	10.3 (3.45–18.41)	0.829
		**Survivors (*N =* 75)**	12.41 (5.93–29)	6.27 (3–12.27)	3.53 (2.26–6.25)	**<0.001**
		*p* **	**<0.001/0.007**			-

* Related-samples Friedman’s two-way analysis of variance by ranks; ** Mann–Whitney U Test (T0 vs. T1 difference/T0 vs. T2 difference between survival groups); *** Repeated-Measures One-Way ANOVA with Greenhouse–Geisser correction; SD = standard deviation; IQR = interquartile range; T0, 1, and 2 = Time 0, 1, and 2; CRP = C-reactive protein; NLR = neutrophil-to-lymphocyte ratio.

## Data Availability

The corresponding author can provide the data used in this study upon reasonable request.

## Abbreviations

The following abbreviations are used in this manuscript:

CHF	Chronic Heart Failure
CKD	Chronic Kidney Diseases
CLD	Chronic Liver Disease
CPOD	Chronic Obstructive Pulmonary Disease
CRP	C-reactive protein
DM	Diabetes mellitus
ESBL	Extended-spectrum beta-lactamase
EUCAST	European Committee on Antimicrobial Susceptibility Testing
HIV	Human immunodeficiency virus
ICU	Intensive care unit
IL-1β	Interleukin-1 beta
IL-6	Interleukin-6
IQR	Interquartile range
KPC	Klebsiella pneumoniae carbapenemase
Ly	Lymphocytes
MALDI-TOF	Matrix-Assisted Laser Desorption/Ionization—Time of Flight Mass Spectrometry
MRSA	Methicillin-Resistant Staphylococcus Aureus
NDM	New Delhi metallo-β-lactamase
NEU, Ne	Neutrophils
NLR	Neutrophil-to-lymphocyte ratio
OXA-48	Oxacillinase 48
PCT	Procalcitonin
RT-PCR	Reverse Transcription Polymerase Chain Reaction
SD	Standard deviation
SIRS	Systemic inflammatory response syndrome
SOFA	Sequential Organ Failure Assessment
T0,1,2	Time 0,1,2
TB	Tuberculosis
TMP/SMX	Trimethoprim/sulfamethoxazole
TNF-α	Tumor necrosis factor-alpha
VRE	Vancomycin-Resistant Enterococci
WBC	White blood cell count
yrs	years
